# Robot-Assisted Hybrid Esophagectomy Is Associated with a Shorter Length of Stay Compared to Conventional Transthoracic Esophagectomy: A Retrospective Study

**DOI:** 10.1155/2017/6907896

**Published:** 2017-12-06

**Authors:** Hans C. Rolff, Rikard B. Ambrus, Mohammed Belmouhand, Michael P. Achiam, Marianne Wegmann, Mette Siemsen, Steen C. Kofoed, Lars B. Svendsen

**Affiliations:** Department of Surgical Gastroenterology, Rigshospitalet, University of Copenhagen, Blegdamsvej 9, 2100 Copenhagen, Denmark

## Abstract

**Aim:**

To compare the peri- and postoperative data between a hybrid minimally invasive esophagectomy (HMIE) and the conventional Ivor Lewis esophagectomy.

**Methods:**

Retrospective comparison of perioperative characteristics, postoperative complications, and survival between HMIE and Ivor Lewis esophagectomy.

**Results:**

216 patients were included, with 160 procedures performed with the conventional and 56 with the HMIE approach. Lower perioperative blood loss was found in the HMIE group (600 ml versus 200 ml, *p* < 0.001). Also, a higher median number of lymph nodes were harvested in the HMIE group (median 28) than in the conventional group (median 23) (*p* = 0.002). The median length of stay was longer in the conventional group compared to the HMIE group (11.5 days versus 10.0 days, *p* = 0.03). Patients in the HMIE group experienced fewer grade 2 or higher complications than the conventional group (39% versus 57%, *p* = 0.03). The rate of all pulmonary (51% versus 43%, *p* = 0.32) and severe pulmonary complications (38% versus 18%, *p* = 0.23) was not statistically different between the groups.

**Conclusions:**

The HMIE was associated with lower intraoperative blood loss, a higher lymph node harvest, and a shorter hospital stay. However, the inborn limitations with the retrospective design stress a need for prospective randomized studies. Registration number is DRKS00013023.

## 1. Introduction

Surgery is the treatment of choice for resectable tumors in the distal esophagus and at the gastroesophageal junction. However, the surgical procedure is associated with a high incidence of postoperative morbidity and mortality with the latter ranging from 2 to 10% [1, 2]. In order to minimize postoperative morbidity and mortality, minimally invasive esophagectomy has been implemented. Minimally invasive surgery diminishes the surgical trauma [3], reduces blood loss and overall hospital stay [4–6], and has comparable oncological results to open surgery [7, 8]. Nevertheless, the conventional minimally invasive surgical methods are challenged by technical aspects, such as two-dimensional view with lack of depth perceptions, long and rigid instruments, and uncomfortable positions for the surgeon [9]. These limitations are less prominent with the implementation of robot-assisted esophagectomy, which allows three-dimensional view and improved articulation of instruments with seven degrees of freedom [10]. Still, data on the clinical effects of robot-assisted esophagectomy are scarce with only few studies published [11, 12].

At our department, a hybrid minimally invasive Ivor Lewis esophagectomy (HMIE), with robot-assisted laparoscopic access to the abdominal cavity and conventional thoracotomy, was implemented during spring 2013. Accordingly, both the HMIE and the conventional Ivor Lewis esophagectomy have been standard procedures since the implementation of the robot-assisted procedure. The aim of this study was to compare the intra- and postoperative data between the two surgical approaches.

## 2. Materials and Methods

### 2.1. Patients

Data from all patients operated for adenocarcinoma in the distal esophagus, at a tertiary referral center in the period from January 1, 2013, to June 1, 2015, were included in the analysis. The patients were retrospectively identified by using the operation codes of The Nordic Medico-Statistical Committee related to resection of distal EC [13]. The presented results from the HMIE group included the learning curve. Initially the operating Robot (daVinci® Si System, Intuitive Surgical Inc., Sunnyvale, CA, USA) was available for 1 day a week; however, resections for distal EC were performed 3 days weekly; thus patients were allocated randomly according to the operation day. However patients that were initially allocated to the HMIE procedure were disqualified if obese (BMI ≥ 35), had a history of previous open abdominal surgery, or were suspected for having a T4-tumor. After two months, the restriction regarding obesity was revised due to good experience with borderline obese cases.

### 2.2. Preoperative Management

All patients followed a standard program after referral to our center. Accordingly, the patients underwent a confirmatory esophagogastroduodenoscopy followed by a contrast enhanced thoracoabdominal computed tomography (CT) scan (or positron emission tomography- (PET-) CT scan if indicated). These examinations were supplemented by an ultrasound examination of the neck and a pulmonary function test, prior to a multidisciplinary conference with the presence of specialists within the fields of surgery, oncology, radiology, pathology, and nuclear medicine. If a patient was considered eligible for surgery, a diagnostic laparoscopy was performed to evaluate the resectability. Prior to definitive surgery, patients with adenocarcinoma were referred for perioperative oncological adjuvant chemotherapy according to the MAGIC-regimen [14].

### 2.3. Surgical Procedures

The conventional transthoracic esophagectomy started with upper laparotomy. Hereafter resectability was again evaluated by ruling out any signs of distant or nonresectable spread. The gastroesophageal junction and the hiatus is hereafter exposed by division of the gastrohepatic and the phrenoesophageal ligaments. The stomach is mobilized at the greater curvature by division of the short gastric vessels and the gastrocolic ligament under the consideration of the right gastroepiploic artery. The stomach is lifted up and the left gastric vessels are identified and divided. The stomach is then mobilized from the lesser curvature to the right gastric artery. This step is followed by Kocher Maneuver. The stomach conduit was then prepared and a pyloromyotomy was performed.

In contrast, the robot-assisted laparoscopy started with insufflation (12 mmHg) with the patient in a supine position, followed by mobilization of the stomach, lymphadenectomy, and division of arteries with the patient in a 13-degree head-up tilt position, as described for the open procedure. After the closure of abdomen (conventional procedure) and desufflation (robot-assisted laparoscopy), the thoracic surgical procedure was identical in both groups; a right thoracotomy at the sixth intercostal space to remove the tumor en bloc along with mediastinal, subcarinal, and paraesophageal lymph nodes (D1+ lymphadenectomy). After resection of the tumor, gastric continuity was reestablished between the esophagus and the remnant corpus part of the stomach, and the surgery was concluded with the placement of a nasogastric tube and a chest tube.

### 2.4. Postoperative Management

All patients followed a standardized postoperative care regimen and were mobilized from day one. Pain was managed using epidural analgesia until the postoperative day three, and patients were individually supplemented with paracetamol, tramadol, or morphine if indicated. During the first week after surgery, patients followed a strict nil-by-mouth regimen and were instead supplied with intravenous nutrition. At the 7th postoperative day, an X-ray with orally administered contrast swallow was performed to evaluate the integrity of the anastomosis, with reintroduction of liquid oral intake if no sign of anastomotic leakage was detected.

### 2.5. Study Design

The present study was a retrospective, nonrandomized, single center evaluation, comparing the conventional with the HMIE. In cases where the HMIE were converted into a conventional open procedure, the results were presented as a conventional procedure.

As this was a retrospective study assessing the treatment quality no ethical approval was required. All patient sensitive data were treated anonymously and were not directly transferable to the individual patient.

### 2.6. Patient Data Registration

All preoperative data regarding demography, comorbidities, height, and weight were retrieved from the electronic patient records. Also, data regarding postoperative morbidity and mortality were registered according to the Clavien-Dindo classification [15] (Table 1), followed by the calculation of the comprehensive complication index (CCI) score [16] using the online tool available at http://www.assessurgery.com/about_cci-calculator/. All events were recorded individually by two investigators and disagreements were settled by discussion within the group. Anastomotic insufficiency was confirmed by either contrast enhanced CT scan/X-ray or by endoscopy and was graded according to Svendsen et al. [17].

### 2.7. Statistics

Descriptive statistics for continuous variables are presented as median (min., max.) and dichotomous variables are presented by the absolute number and the percentage of positives. Continuous variables were compared using Mann-Whitney* U* test, while *χ*^2^-test was used for categorical variables.

The statistical analyses were performed using the SPSS-software (IBM SPSS statistics for Windows, Version 22.0. Armonk, NY). *p* values < 0.05 were considered significant. The comparison of long-term survival between groups was conducted through a Log-Rank test.

## 3. Results

During the period of January 1, 2013, to June 1, 2015, a total of 216 patients were included in the statistical analysis, with 160 procedures performed with the conventional approach and 56 with the HMIE approach. Two cases were converted due to obesity and severe adhesions.

There were no differences regarding age and gender distribution, body mass index (BMI), or ASA-scores between the two groups (Table 2).

A significantly lower total blood loss was found in the HMIE group compared to the conventional group (600 ml versus 200 ml, *p* < 0.001). Also, the median number of harvested lymph nodes was significantly higher in the HMIE group (median 28) than in the conventional group (median 23) (*p* = 0.002). In contrast, neither the time of general anaesthesia, operating time, nor the total procedure time was statistically different between the groups.

The median length of hospital stay was significantly longer in the conventional group compared to the HMIE group (11.5 days versus 10.0 days, *p* = 0.03). There were no differences in postoperative complications between the conventional and the HMIE group regarding the proportion of patients experiencing one or more complications of grade I or higher (76% versus 65%, *p* = 0.12), just as no difference in CCI score (12.2 versus 20.9, (*p* = 0.12)) was found. The proportion of patients in the HMIE group which experienced one or more complications of grade II or higher was significantly lower than in the conventional group (39% versus 57%, *p* = 0.03). The rate of anastomotic insufficiency was identical (7%) (*p* = 1.00), and there were no significant differences regarding the 30- and 90-day mortality between the groups.

The rate of all pulmonary complications is shown in Table 3 and shows no statistical differences between the two groups, both regarding the sum of pulmonary complications and when looking isolated at the severe pulmonary complications. However there was a trend towards fewer severe complications in the HMIE groups, but this finding was not statistically significant.

There was no statistically significant difference between the two groups regarding the long-term survival (*p* = 0.7).

## 4. Discussion

In the present study, we found that patients operated with the HMIE approach had a lesser surgical blood loss, had more lymph nodes harvested, and had a shorter hospital stay compared to patients undergoing conventional Ivor Lewis esophagectomy. Furthermore, the HMIE group experienced fewer grade ≥II complications than the conventional group.

The abovementioned perioperative benefits regarding blood loss and lymph node harvest related to the HMIE approach could reflect that the robot assistance offers a more precise and refined dissection phase compared to the conventional approach. This has benefits, as it has been proposed that blood transfusions are associated to a poorer long-term outcome in cancer patients [18] and are thus important to avoid. The impact of the extent of lymph node resection on long-term survival is much debated. Some papers advocate that the number of removed lymph nodes is an independent prognostic marker [19]. Other reports state that increasing the number of harvested lymph nodes does not per se offer any improvement in the survival [20]. However, the number of metastatic nodes removed and increasing positive-to-negative node ratio were strongly negatively associated to survival [20]. Thus an increased lymph node harvest leads to a better staging. In the present series the increased number of harvested lymph nodes in the HMIE group did not translate into an improved survival, indicating that there is little or no impact on survival. The presented number of harvested lymph nodes in this series is the total amount, and the lymph nodes therefore originate from both the thorax and abdomen. As the thoracic approach was identical it is plausible that the difference in lymph node harvest was due to the different abdominal approaches.

The findings, regarding the postoperative complications, were not entirely clear. No difference in the CCI score between the groups was found; however, a significantly lower proportion of the patients in the HMIE group experienced one or more grade II complications, compared to the conventional group in the current study. These findings are in accordance with the available literature, where the majority of studies report no major differences between the minimally invasive and conventional approaches [21]. Accordingly, some studies report fewer complications with a minimally invasive approach [22, 23], but reports have also indicated that the minimally invasive approach was associated with a higher frequency of acute reoperation [24]. In this study, there was no difference in the pattern of grade II complications between the groups, and thus no apparent explanation for this difference. There was no difference between groups regarding the more severe grade ≥III complications.

In general, the quantification of postoperative complications is semiquantitative by nature, making direct comparison between studies difficult. The Clavien-Dindo grading system does offer some standardization, but different interpretations of the grading system, especially in the low-grade complication range, are likely to occur. Due to these difficulties in getting an objective measure, surrogate markers for the quantification of the complexity of the postoperative course could be used. One such parameter is the total length of hospital stay, which in this study was shorter in the HMIE group compared to the conventional group. This fact may have important economic implications, as a shortening of the admission time may level out the higher costs associated with the robot-assisted procedure. This benefit has been reported for gynecological and urological cancers [25, 26]. More interestingly, it is conceivable that the length of stay and rate of postoperative complications may be further reduced by introducing MIS in the thoracic part of the procedure.

Currently, no consensus regarding the role of MIS in the surgical treatment of upper gastrointestinal cancers exists. A consensus is difficult to define, since the surgical strategies for treating upper gastrointestinal cancers cover a very heterogeneous group of procedures, both regarding the type of access, that is, laparoscopy and robot-assisted laparoscopy, and whether or not these modalities should be applied in the abdomen and/or the thorax. Furthermore, the study designs in previous studies investigating the role of MIS have been suboptimal, with the vast majority being retrospective with small patient volume and with great variation of the surgical techniques among the studies. This aspect is also a limitation in the present study, as this was conducted retrospectively at a single center. This only highlights the need for large prospective randomized trials. Such trials have been registered [7, 27, 28]. Trials comparing the HMIE with the conventional Ivor Lewis esophagectomy especially are relevant due to the similar surgical techniques between the studies [27, 29]. Messager et al. showed significantly reduced perioperative mortality when comparing the HMIE with the conventional procedure [29]. This feat was achieved without a higher rate of reoperation, which had been a concern in previous reports. However, the rate and type of postoperative complications were not reported in the study [29]. Data from the MIRO-trial [27] do show that the rate of pulmonary complications was significantly decreased in the hybrid group. We were unable to reproduce these results; however, there was a trend towards fewer severe pulmonary complications in the HMIE group. Most significantly we found that the length of stay was reduced for the HMIE group. The findings from our study in combination with randomized HMIE studies indicate that HMIE could offer important advantages and may be the future standard surgical strategy for patients with malignant tumors in the distal esophagus.

## 5. Conclusion

This study shows that HMIE was associated with a significantly reduced intraoperative blood loss, a higher number of harvested lymph nodes, and a shorter hospital stay. Whether this is solely due to the less invasiveness of the HMIE compared to conventional Ivor Lewis esophagectomy needs to be investigated further and the possible advantages must be confirmed in a prospective randomized setting.

## Figures and Tables

**Table 1:** Clavien-Dindo classification of postoperative complications.

Grade I	Any deviation from the normal postoperative course without the need for pharmacological treatment or surgical, endoscopic, and radiological interventions. Allowed therapeutic regimens are drugs as antiemetics, antipyretics, analgetics, diuretics, electrolytes, and physiotherapy. This grade also includes wound infections opened at the bedside.

Grade II	Requiring pharmacological treatment with drugs other than such allowed for grade I complications. Blood transfusions and total parenteral nutrition are also included.

Grade III	Requiring surgical, endoscopic, or radiological intervention.
(i) IIIa	Intervention not under general anesthesia.
(ii) IIIb	Intervention under general anesthesia

Grade IV	Life-threatening complications (including CNS complications) requiring IC/ICU management.
(i) IVa	Single organ dysfunction (including dialysis).
(ii) IVb	Multiorgan dysfunction.

Grade V	Death of the patient.

**Table 2:** Comparison of pre-, peri-, and postoperative data between cohorts.

	Conventional (*n* = 160)	HMIE (*n* = 56)	*p* value
Age^*∗*^	65 (28–88)	66 (39–86)	0.65

Gender			
(i) Male	125 (78%)	50 (88%)	
(ii) Female	35 (22%)	6 (12%)	0.12

BMI^*∗*^	26.6 (15.6–43.7)	25.8 (18.8–31.2)	0.19

ASA-score			
(i) ASA 1 (%)	41 (26%)	17 (30%)	0.73
(ii) ASA 2 (%)	80 (50%)	28 (50%)	1
(iii) ASA 3 (%)	39 (24%)	12 (21%)	0.72
(iv) ASA 4 (%)	0 (0%)	1 (2%)	

Operating time^*∗*^ (minutes)	248 (100–420)	232 (174–800)	0.2

Blood loss^*∗*^ (ml)	600 (100–4400)	200 (50–1970)	<0.001

Harvested lymph nodes^*∗*^	23 (11–60)	28 (15–61)	0.002

Length of stay^*∗*^ (days)	11.5 (8–101)	10 (8–69)	0.03

CCI-score	20.9 (0–100)	12.2 (0–100)	0.22

Complications^*∗∗*^			
(i) ≥Grade I complications (%)	122 (76%)	37 (65%)	0.12
(ii) ≥Grade II complications (%)	91 (57%)	22 (39%)	0.02
(iii) ≥Grade III complications (%)	51 (32%)	14 (25%)	0.32

Anastomotic insufficiency	11 (7%)	4 (7%)	1

30-day mortality (%)	3 (2%)	0 (0%)	0.57

90-day mortality (%)	5 (3%)	3 (5%)	0.43

HMIE: hybrid minimally invasive esophagectomy; CCI: comprehensive complication index. These scores are generated from http://www.assessurgery.com/about_cci-calculator/; ^*∗*^continuous covariates are presented with median and minimum and maximum values; ^*∗∗*^complications are graded according to the Clavien-Dindo score. The numbers represents the proportion of patients experiencing one or more complications of at least the grade indicated in the table.

**Table 3:** Pulmonary complications.

	Conventional (*n* = 160)	HMIE (*n* = 56)	*p* value
All pulmonary complications	51% (81/160)	43% (24/56)	0.32
Severe pulmonary complications^*∗*^	38% (41/160)	18% (10/56)	0.24

^*∗*^Severe respiratory complications are defined as grade III or higher on the Clavien-Dindo score.
